# Effects of Winter Cover Crops Straws Incorporation on CH_4_ and N_2_O Emission from Double-Cropping Paddy Fields in Southern China

**DOI:** 10.1371/journal.pone.0108322

**Published:** 2014-10-01

**Authors:** Hai-Ming Tang, Xiao-Ping Xiao, Wen-Guang Tang, Ke Wang, Ji-Min Sun, Wei-Yan Li, Guang-Li Yang

**Affiliations:** Hunan Soil and Fertilizer Institute, Changsha, PR China; Tennessee State University, United States of America

## Abstract

Residue management in cropping systems is believed to improve soil quality. However, the effects of residue management on methane (CH_4_) and nitrous oxide (N_2_O) emissions from paddy field in Southern China have not been well researched. The emissions of CH_4_ and N_2_O were investigated in double cropping rice (*Oryza sativa* L.) systems with straw returning of different winter cover crops by using the static chamber-gas chromatography technique. A randomized block experiment with three replications was established in 2004 in Hunan Province, China, including rice–rice–ryegrass (*Lolium multiflorum* L.) (Ry-R-R), rice–rice–Chinese milk vetch (*Astragalus sinicus* L.) (Mv-R-R) and rice–rice with winter fallow (Fa-R-R). The results showed that straw returning of winter crops significantly increased the CH_4_ emission during both rice growing seasons when compared with Fa-R-R. Ry-R-R plots had the largest CH_4_ emissions during the early rice growing season with 14.235 and 15.906 g m^−2^ in 2012 and 2013, respectively, when Ry-R-R plots had the largest CH_4_ emission during the later rice growing season with 35.673 and 38.606 g m^−2^ in 2012 and 2013, respectively. The Ry-R-R and Mv-R-R also had larger N_2_O emissions than Fa-R-R in both rice seasons. When compared to Fa-R-R, total N_2_O emissions in the early rice growing season were increased by 0.05 g m^−2^ in Ry-R-R and 0.063 g m^−2^ in Mv-R-R in 2012, and by 0.058 g m^−2^ in Ry-R-R and 0.068 g m^−2^ in Mv-R-R in 2013, respectively. Similar result were obtained in the late rice growing season, and the total N_2_O emissions were increased by 0.104 g m^−2^ in Ry-R-R and 0.073 g m^−2^ in Mv-R-R in 2012, and by 0.108 g m^−2^ in Ry-R-R and 0.076 g m^−2^ in Mv-R-R in 2013, respectively. The global warming potentials (GWPs) from paddy fields were ranked as Ry-R-R>Mv-R-R>Fa-R-R. As a result, straw returning of winter cover crops has significant effects on increase of CH_4_ and N_2_O emission from paddy field in double cropping rice system.

## Introduction

With the current rise in global temperatures, numerous studies have focused on greenhouse gases (GHG) emissions [1]–[3]. Agriculture production is an important source of GHG emission [4]. In addition to carbon dioxide (CO_2_), methane (CH_4_) and nitrous oxide (N_2_O) play important roles in global warming. The global warming potentials (GWPs) of CH_4_ and N_2_O are 25 and 298 times that of CO_2_ in a time horizon of 100 years, respectively [5]. The concentrations of CH_4_ and N_2_O in the atmosphere are estimated to be increasing at the rates of 1% and 0.2–0.3% per year [6]. In addition to industrial emissions, farmland is another important source of atmospheric GHG [7]–[10]. Numerous results indicate that rice (*Oryza sativa* L.) paddy field is a significant source of CH_4_ and N_2_O emissions [10], [11]. The anaerobic conditions in wetland rice field are favorable for fostering CH_4_ emission [12]. Thus, the characteristics of CH_4_ and N_2_O emissions from paddy field and the reduction of emission have received attentions from scientists.

A considerable number of studies have shown that some farm operations can influence CH_4_ and N_2_O emission. For example, cropping system, crop type, water and nitrogen (N) management, organic matter application and tillage can regulate CH_4_ and N_2_O emission [13]–[15]. Tillage and crop straws retention have a great influence on CH_4_ and N_2_O emission through the changes of soil properties (e.g., soil porosity, soil temperature and soil moisture, etc.) [16]–[17]. In paddy soils, CH_4_ is produced by archaea bacteria during the anaerobic degradation of organic matter and oxidized by methanotrophic bacteria [18]. Incorporation of organic material into soil can enhance the number and activity of archaea bacteria [19] and provide large amounts of active organic substrate for CH_4_ production [20]. Soil amendment with organic material, such as crop straw [21] and green manure incorporation [22], has been well estimated to promote CH_4_ emission in paddy fields. Biogenic N_2_O production originates from nitrification and denitrification [23], which are processes involving microorganisms in the soil. N_2_O flux in paddy fields was small in flooding condition, but peaked after drainage [24]. Some studies have indicated that the cropping system of winter fallow with cover crops has advantages of promoting soil quality, enhancing nutrient utilization, increasing crop yield, reducing soil erosion and chemical runoff, and inhibiting weed growth in paddy field [25]–[26].

Winter cover crops, which are grown during an otherwise fallow period, are a possible means of improving nutrient dynamics in the surface layer of intensively managed cropping systems. Chinese milk vetch (*Astragalus sinicus* L.) and ryegrass (*Lolium multiflorum* L.) are the main winter cover crops in Southern China. Growing these cover crops with straw mulching in the winter season after late rice harvest and incorporating them into soil as green manure before early rice transplanting next year is a traditional practice as well as rice straw incorporation. Hermawan and Bomke [26] suggested that growing winter cover crops such as annual ryegrass may protect aggregate breakdown during winter and result in a better soil structure after spring tillage, as opposed to leaving soil bare. Other potential benefits of winter cover crops are the prevention of nitrate leaching [27]; weed infestation [28]; and improvement of soil water retention, soil organic matter content and microbial activity [29]. Returning of crop straws have been suggested to improve overall soil conditions, reduce the requirement for N fertilizers and support sustainable rice productivity.

In recent years, many researches have studied the effects of winter cover crops on soil physical properties and crop productivity, methane emission, N availability and nitrogen surplus [30]–[32]. However, relatively few studies related to CH_4_ and N_2_O emissions and yields under different double cropping rice systems with different winter cover crops have been conducted in double–cropping paddy field in Southern China. Monitoring CH_4_ and N_2_O emissions of different winter cover crops–double cropping rice cultivation modes is important to maintain soil productivity, increase carbon (C) storage, and regulate the greenhouse effects. Therefore, the objectives of this research were: (1) to quantify CH_4_ and N_2_O emissions from paddy field and grain yield under different winter cover crops and double cropping rice systems, (2) to evaluate the GWPs of different winter cover crops–double cropping rice treatments in southern China.

## Materials and Methods

### Experimental site

**Figure 1:**
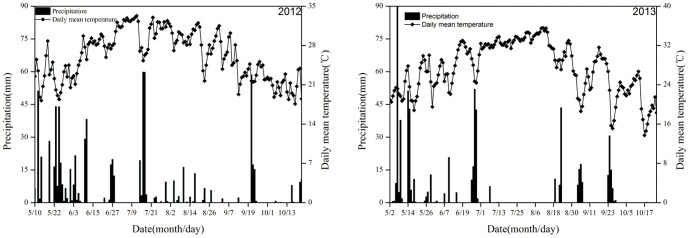
Daily precipitation and mean temperature at the study site in 2012 and 2013.

The experiment was initiated in winter 2004 at the experimental station of the Institute of Soil and Fertilizer Research, Hunan Academy of Agricultural Sciences, China (28°11′58″ N, 113°04′47″ E). The typical cropping system in this area is double cropping rice. The soil type is a Fe–accumuli–Stagnic Anthrosol derived from Quaternary red clay (clay loam). The characteristics of the surface soil (0–20 cm) in 2004 are as follows: pH 5.40, soil organic carbon (SOC) 13.30 g kg^−1^, total N 1.46 g kg^−1^, available N 154.5 mg kg^−1^, total phosphorous (P) 0.81 g kg^−1^, available P 39.2 mg kg^−1^, total potassium (K) 13.0 g kg^−1^, and available K 57.0 mg kg^−1^. All these data were tested before the experiment in 2004. This region has the subtropical monsoonal humid climate with a long hot period and short cold period. The average annual precipitation is approximately 1500 mm and the annual mean temperature is 17.1°C, the annual frost-free period is approximately from 270 days to 310 days. The daily precipitation and mean temperature data during the early and late rice growing season during 2012–2013 are presented in Fig. 1. The cropping system was that the early rice rotated with the late rice, and then planted winter cover crops till the next year's early rice transplanting.

### Experimental design and field management

A randomized block experiment with three replications was established in 2004, and this study was conducted from 2012 to 2013. The experiment included three cropping systems: rice–rice–ryegrass (Ry-R-R), rice–rice–Chinese milk vetch (Mv-R-R), and rice–rice with winter fallow (Fa-R-R). The plot area was 1.1 m^2^ (1 m × 1.1 m). After winter cover crops harvested, a moldboard plow was used to incorporate part of the crop straw into soil: both the ryegrass and Chinese milk vetch straw returned was 22500.0 kg ha^−1^. All the plots were plowed once to a depth of 20 cm by using a moldboard plow 15 d before rice seedling transplanting. The early rice variety (*Oryza sativa* L.) Lingliangyou 211 and late rice variety (*Oryza sativa* L.) Fengyuanyou 299 were used as the materials in 2012 and 2013. One-month-old seedlings were transplanted with a density of 150,000 plants ha^−1^ (one seed per 16 cm × 16 cm) and 2–3 plants per hill. Gramoxone (paraquat) was applied to control weeds at 2 d before rice transplantation. The basal fertilizer of the early and late rice was applied at the rate of 150.0 kg N ha^−1^ and 180.0 kg N ha^−1^ as urea (60% for basal; 40% for top–dressed at the tillering stage), 75.0 kg P_2_O_5_ ha^−1^ as diammonium phosphate and 120.0 kg K_2_O ha^−1^ as potassium sulfate. The different treatments during early and late rice season and field management were presented in Table 1.

**Table 1:** Management practices of different cropping systems.

Crop	Date (month/day)		Field management
	2012	2013	
Early rice	4/12	4/5	Sowing and seedling raising
	5/9	5/1	Paddy tillage
	5/10	5/2	Transplanting (16 cm×16 cm)
	5/18	5/10	Urea were applied at 130.0 kg ha^−1^ for top–dressed at tillering
	6/7–6/15	5/27–6/5	Drained out water and dried the soil at maximum tillering stage
	6/16–7/13	6/6–7/13	Wetting–drying alternation irrigation
	7/18	7/18	Grains were harvested
Late rice	6/25	6/27	Sowing and seedling raising
	7/21	7/19	Paddy tillage (The rate of early rice straw returning was 4 500.0 kg ha^−1^)
	7/22	7/20	Transplanting (16 cm×16 cm)
	7/30	7/28	Urea were applied at 156.5 kg ha^−1^ for top–dressed at tillering
	8/20–8/27	8/16–8/26	Drained out water and dried the soil at maximal tillering stage
	8/28–10/17	8/27–10/19	Wetting–drying alternation irrigation
	10/22	10/25	Grains were harvested

### Collection and measurement of CH_4_ and N_2_O

CH_4_ and N_2_O emitted from paddy field were collected using the static chamber–GC technique at 9:00–11:00 in the morning during the early and late rice growing season. The chamber (50 cm × 50 cm × 120 cm) was made of 5 mm PVC board with a PVC base. The base had a groove in the collar, in which the chamber could be settled. The chamber base was inserted into soil about 5 cm in depth with rice plant growing inside the base. The groove was 1 cm below flooded water, and the chamber was settled into the groove of the collar with water to prevent leakage and gas exchange. The chamber contained a small fan for stirring air, a thermometer sensor, and a trinal–venthole. From the second day after transplanting of early or late rice, gases were sampled weekly. Before sampling, the fan in the chamber started working to allow an even mix of air before extracting the air with a 50 ml injector at 0, 10, 20, and 30 min after closing the box. The air samples were transferred into 0.5 L sealed sample bags by rotating trinal venthole.

The quantities of CH_4_ and N_2_O emission were measured with a gas chromatograph (Agilent 7890A) equipped with flame ionization detector (FID) and electron capture detector (ECD). Methane was separated using 2 m stainless-steel column with an inner diameter of 2 mm 13XMS column (60/80 mesh), with FID at 200°C. Nitrous oxide was separated using a 1 m stainless-steel column with an inner diameter 2 mm Porapak Q (80/100 mesh) and ECD at 330°C.

### Data analysis

Fluxes of CH_4_ and N_2_O were calculated with the following equation [33]:
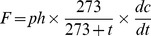



Where, F is the CH_4_ flux (mg m^−2^ h^−1^) or N_2_O flux (µg m^−2^ h^−1^); *T* is the air temperature (°C) inside the chamber; *ρ* is the CH_4_ or N_2_O density at standard state (0.714 kg m^−3^ for CH_4_ and 1.964 kg m^−3^ for N_2_O); *h* is the headspace height of the chamber (m); and d*c*/d*t* is the slope of the curve of gas concentration variation with time.

The total emissions of CH_4_ and N_2_O were sequentially computed from the emissions between every 2 adjacent intervals of the measurements, based on a non–linear, least–squares method of analysis [34], [35].

GWPs is defined as the cumulative radiative forcing both direct and indirect effects integrated over a period of time from the emission of a unit mass of gas relative to some reference gas. Carbon dioxide was chosen as this reference gas. The GWPs conversion parameters of CH_4_ and N_2_O (over 100 years) were adopted with 25 and 298 kg ha^−1^ CO_2_-equivalent [5].

### Statistical analysis

Data presented herein are means of 3 replicates in each treatment. All data were expressed as mean ± standard error. The data were analyzed as a randomized complete block, using the PROC ANOVA procedure of SAS [36]. Mean values were compared using the least significant difference (LSD) test, and a probability value of 0.05 was considered to indicate statistical significance.

## Results

### Characteristics of CH_4_ emission flux from early and late rice fields

**Figure 2:**
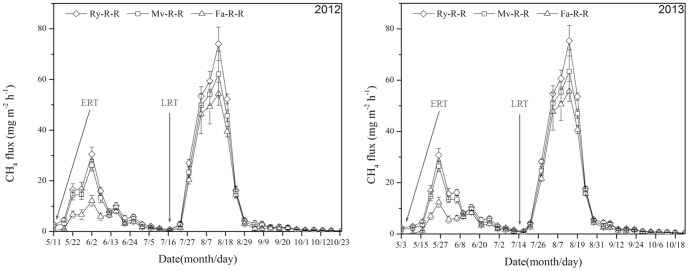
Effects of winter cover crops on CH_4_ flux in early and late rice fields in 2012 and 2013. Ry-R-R: rice–rice–ryegrass cropping system; Mv-R-R: rice–rice–Chinese milk vetch cropping system; Fa-R-R: rice–rice cropping system with winter fallow. ERT: early rice transplanting; LRT: late rice transplanting. CH_4_ emission rate is the mean of values measured within each treatment (n = 3).

In the early rice season, the curve of CH_4_ flux was low when early rice was newly transplanted, but increased quickly until the first peak about 2 weeks after transplanting, and then dramatically declined to a low level with relative stability with the second small peak appeared at 36 and 35 d after transplanting in 2012 and 2013, respectively (Fig. 2). The gradual increase of CH_4_ emission after transplanting resulted from the decomposition of organic matter and the growth of rice. The second peak was mainly because of the continuous decomposition of organic matter under high temperature. In the early rice season, the CH_4_ flux values were significantly different among treatments with the order of Ry-R-R>Mv-R-R>Fa-R-R (*P<*0.05) (Fig. 2).

Methane emission in the late rice growing season mainly focused at tillering stage, and the peak value of CH_4_ flux was observed at 23 and 24 d after transplanting in all treatments in 2012 and 2013, respectively. Then, the emission rate dramatically decreased to a low and stable level, especially from field drainage to harvest. The order of treatments in CH_4_ emission was Ry-R-R>Mv-R-R>Fa-R-R (Fig. 2).

### Characteristics of N_2_O emission flux from early and late rice fields

**Figure 3:**
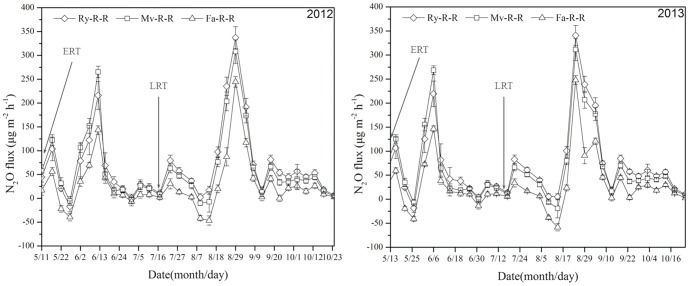
Effects of winter cover crops on N_2_O flux in early and late rice fields in 2012 and 2013. Ry-R-R: rice–rice–ryegrass cropping system; Mv-R-R: rice–rice–Chinese milk vetch cropping system; Fa-R-R: rice–rice cropping system with winter fallow. ERT: early rice transplanting; LRT: late rice transplanting. N_2_O emission rate is the mean of values measured within each treatment (n = 3).

The peak flux N_2_O was emitted when the field was drained. Meanwhile, part of N_2_O was emitted during wetting–drying alternation irrigation period. The first peak value of N_2_O flux appeared at 7 and 15 d after transplanting in all treatments in 2012 and 2013, respectively, and then decreased. The order among treatments was Mv-R-R>Ry-R-R>Fa-R-R during the period from transplanting to field drainage, and Ry-R-R>Mv-R-R>Fa-R-R during wetting–drying alternation period. The N_2_O flux in early rice paddy reached the highest peak at 32 and 35 d after transplanting in 2012 and 2013, respectively (Fig. 3).

In the late rice growing season, N_2_O emission increased from field drainage to full heading stage, and mainly focused at booting stage. The order of N_2_O emission fluxes among different treatments was Ry-R-R>Mv-R-R>Fa-R-R in the late rice growing season. In 2012, the average N_2_O fluxes in the late rice growing season were 78.718 µg m^−2^ h^−1^ in Ry-R-R, 64.928 µg m^−2^ h^−1^ in Mv-R-R, and 32.275 µg m^−2^ h^−1^ in Fa-R-R. In 2013, the average N_2_O fluxes in the late rice growing season were 81.453 µg m^−2^ h^−1^ in Ry-R-R, 67.662 µg m^−2^ h^−1^ in Mv-R-R, and 34.623 µg m^−2^ h^−1^ in Fa-R-R (Fig. 3).

### Total CH_4_ and N_2_O emission from paddy fields in the growing durations of early and late rice

In the early rice growing season, the total CH_4_ emissions of Ry-R-R and Mv-R-R were significantly higher than Fa-R-R (*P<*0.05), and the order of treatments was Ry-R-R>Mv-R-R>Fa-R-R (Table 2). The straws of winter cover crops incorporated into soil provided favorable soil condition and sufficient substance to be decomposed in the early rice season; therefore, the CH_4_ emission quantities in straw returning treatments were higher than Fa-R-R (*P<*0.05). In 2012, the total CH_4_ emissions from paddy fields during late rice entire growing season were 35.673 g m^-2^ in Ry-R-R, 31.542 g m^−2^ in Mv-R-R, 27.874 g m^−2^ in Fa-R-R. In 2013, the total CH_4_ emissions from paddy fields during late rice whole growing season were 38.606 g m^−2^ in Ry-R-R, 34.358 g m^−2^ in Mv-R-R, 30.550 g m^−2^ in Fa-R-R. The order of treatments in total CH_4_ emission was Ry-R-R>Mv-R-R>Fa-R-R (Table 2).

**Table 2:** Effects of winter cover crops on CH_4_ and N_2_O emission from rice fields during whole growing season of early and late rice (g m^−2^).

Year	Treatment	CH_4_			N_2_O		
		Early rice	Late rice	Total	Early rice	Late rice	Total
2012	Ry-R-R	14.235±0.411a	35.673±1.030a	49.908±1.441a	0.088±0.003a	0.176±0.05a	0.264±0.008a
	Mv-R-R	12.092±0.349b	31.542±0.912b	43.634±1.260b	0.101±0.003b	0.145±0.04b	0.246±0.007a
	Fa-R-R	6.732±0.194c	27.874±0.805c	34.606±0.999c	0.038±0.001c	0.072±0.02c	0.110±0.003b
2013	Ry-R-R	15.906±0.459a	38.606±1.115a	54.512±1.574a	0.100±0.003b	0.188±0.006a	0.288±0.008a
	Mv-R-R	13.523±0.390b	34.358±0.992b	47.882±1.382b	0.110±0.003a	0.156±0.005b	0.266±0.008a
	Fa-R-R	7.535±0.218c	30.550±0.882c	38.085±1.099c	0.042±0.002c	0.080±0.002c	0.122±0.004b

Ry-R-R: rice–rice–ryegrass cropping system; Mv-R-R: rice–rice–Chinese milk vetch cropping system; Fa-R-R: rice–rice cropping system with winter fallow.

Values are presented as mean ± SE (n  =  3). Means in each column with different letters are significantly different at the *P* <0.05 level.

Compared to Fa-R-R, the other treatments increased total N_2_O emissions in the early rice growing season, and the N_2_O emissions increased by 0.05 g m^−2^ (131.58%) in Ry-R-R and 0.063 g m^−2^ (165.79%) in Mv-R-R in 2012, and by 0.058 g m^−2^ (138.1%) in Ry-R-R and 0.068 g m^−2^ (161.90%) in Mv-R-R in 2013, respectively. Similar results were observed in the late rice growing season in 2012, the total N_2_O emissions increased by 0.104 g m^−2^ (144.44%) in Ry-R-R and 0.073 g m^−2^ (101.39%) in Mv-R-R. And the total N_2_O emissions increased by 0.108 g m^−2^ (135.00%) in Ry-R-R and 0.076 g m^−2^ (95.00%) in Mv-R-R in 2013 (Table 2).

The emissions of CH_4_ and N_2_O were closely related to farming system, soil type, climate, and field management practices. Ry-R-R and Mv-R-R had larger total CH_4_ emissions than Fa-R-R in the double rice growing season (*P<*0.05). Ry-R-R had the largest total N_2_O emissions in the double rice growing season with the quantities of 0.264 g m^−2^ in 2012, and 0.288 g m^−2^ in 2013, respectively (Table 3).

**Table 3:** Double rice grain yield, global warming potentials (GWPs) of CH_4_ and N_2_O and per yield GWPs from rice fields under different cropping patterns.

Year	Treatment	CH_4_ emission	N_2_O emission	GWPs of CH_4_	GWPs of N_2_O	GWPs of CH_4_ and N_2_O	Double rice grain yield	Per yield GWPs CO_2_
		(g m^−2^)	(g m^−2^)	(kg CO_2_-eq ha^−1^)	(kg CO_2_-eq ha^−1^)	(kg CO_2_-eq ha^−1^)	(kg ha^−1^)	(kg kg ^−1^)
2012	Ry-R-R	49.908±1.441a	0.264±0.008a	12494.38±360.68a	787.41±22.73a	13281.79±383.41a	13800.23±398.38a	0.96±0.03a
	Mv-R-R	43.634±1.260b	0.246±0.007a	10923.85±315.34b	733.58±21.18a	11657.44±336.52b	15089.30±435.59a	0.77±0.02b
	Fa-R-R	34.606±0.999c	0.110±0.003b	8663.66±250.10c	329.46±9.51b	8993.12±259.61c	14359.00±414.51a	0.63±0.02c
2013	Ry-R-R	54.512±1.574a	0.288±0.008a	13646.99±393.95a	859.81±24.82a	14506.80±418.76a	14738.87±425.47a	0.98±0.03a
	Mv-R-R	47.882±1.382b	0.266±0.008a	11987.20±346.04b	793.53±22.91a	12780.73±368.95b	14896.57±430.03a	0.86±0.02b
	Fa-R-R	38.085±1.099c	0.122±0.004b	9534.57±275.24c	364.64±10.53b	9899.22±285.77c	13625.16±322.60a	0.73±0.02c

Ry-R-R: rice–rice–ryegrass cropping system; Mv-R-R: rice–rice–Chinese milk vetch cropping system; Fa-R-R: rice–rice cropping system with winter fallow.

Values are presented as mean ± SE (n  =  3). Means in each column with different letters are significantly different at the *P*<0.05 level.

### Global warming potentials of CH_4_ and N_2_O

GWPs is an indicator to reflect the relative radioactive effect of a greenhouse gas, and the GWPs of CO_2_ is defined as 1. In this study, the GWPs of CH_4_ and N_2_O from double cropping paddy fields varied with different winter cover crops, and the trend showed as Ry-R-R>Mv-R-R>Fa-R-R. In 2012, Ry-R-R had the largest GWPs (13281.79 kg CO_2_–eq ha^−1^) of total CH_4_ and N_2_O from double cropping paddy fields, followed by Mv-R-R (11657.44 kg CO_2_–eq ha^−1^), and Fa-R-R had the lowest GWPs of total CH_4_ and N_2_O (8993.12 kg CO_2_–eq ha^−1^). In 2013, Ry-R-R had the largest GWPs (14506.80 kg CO_2_–eq ha^−1^) of total CH_4_ and N_2_O from double cropping paddy fields, followed by Mv-R-R (12780.73 kg CO_2_–eq ha^−1^), and Fa-R-R had the lowest GWPs of total CH_4_ and N_2_O (9899.22 kg CO_2_–eq ha^−1^). According to GWPs, CH_4_ from double cropping paddy fields had greater contribution to global warming than N_2_O (Table 3).

Double rice grain yield of Mv-R-R was the highest, the lowest was Fa-R-R (Table 3). We also estimated per yield GWPs which was calculated as GWPs divided by rice grain yield. As is shown in Table 3, per yield GWPs of Ry-R-R was significantly higher than Mv-R-R and Fa-R-R (*P<*0.05), and the lowest was Fa-R-R.

## Discussion

### CH_4_ emission

Methane emission is complex processes including production, oxidation, and emission. Chidthaisong et al. [37] reported that the highest CH_4_ peaks were observed at flowering and heading stages, which could be related to the development of intense reducing conditions in the rice rhizosphere. In this study, we found that CH_4_ emission was low in paddy fields after transplanting during early rice–growing season, and increased with the decomposition of organic matters and growth of rice. In addition, CH_4_ emission was influenced by soil temperature and soil redox potential (Eh). Yu et al. [38] reported that CH_4_ emission showed an exponential decrease by an Eh increase. In this study, the CH_4_ flux and total CH_4_ emission from paddy fields during the early and late rice growing season were much larger in Ry-R-R and Mv-R-R compared to Fa-R-R, which was similar to the result by Lee et al. [22]. The reasons for above result may be: first, microbial activities were improved after returning straws of winter cover crops into the soil due to the supplements of carbon source and energy for microbial activities to accelerate consumption of soil oxygen and decrease of soil Eh; second, methanogens became active due to the large quantities of C source, which provided reactive substrate for CH_4_ emission from paddy fields. In the early rice growing season, the order of CH_4_ flux and total CH_4_ emission from paddy fields varied among treatments, which were highly related to the returning straw type, and straw decomposition rate. During the late rice growing season, the CH_4_ emission increased gradually with the decomposition of organic matters and growth of rice after transplanting, and reach the peak value at tillering stage in all treatments. However, CH_4_ emissions in both rice seasons were reduced in a large extent after field drying, because (1) soil aeration was improved during this period, and the activities of methanogens were restricted; and (2) the physiological activity of rice plant decreased, thereby limiting the ability for transportation and emission of CH_4_ [39].

Although straw returning helps to maintain soil fertility and protect environment, but it enhances CH_4_ emission simultaneously. Pandey et al. [12] showed that CH_4_ emission was positively related to straw returning amount under permanent flooding condition, whereas N_2_O emission had a reverse relationship with the amount of straw returning. In this study, we found that CH_4_ flux in the late rice growing season was much higher than that in the early rice growing season, and peak appeared earlier. As straws of early rice (4500 kg ha^−1^) returned to field before transplanting of late rice, the paddy soil of late rice was under anoxic condition after transplanting, which was favorable for CH_4_ production and emission. Temperature was the major reason for the differences in the CH_4_ emission pattern between the early and the late rice season. Soil temperature had a predictive functional relationship with CH_4_ emission. Zhang et al. [40] reported that there was a strong positive correlation between CH_4_ emission and soil temperature. In this experimental area, the late rice season was the hottest time in summer (Fig. 1). Therefore, high temperatures enhanced the decomposition of crop straws in the moist environment. In contrast to the warm temperatures of the late rice season, the air temperatures of the early rice season were lower, which resulted in slower crop straws decomposition and little CH_4_–substrate. Hence, these differences in weather factors (e.g., temperature) resulted in the different characteristics of CH_4_ between the early and the late rice seasons. However, there were significantly differences among treatments although they had similar trends. This indicated that CH_4_ flux and emission from paddy fields were affected by different winter cover crops.

### N_2_O emission

The emissions of N_2_O are closely related to soil moisture, oxygen, temperature, content of soil organic matter and pH [4], [11], [17]. Great positive interaction has been reported between N_2_O emission and green manure or chemical nitrogen fertilizer in early rice growing season [41]. In this study, we found that N_2_O emission in the early rice growing season focused in the period of field drainage, and the Ry-R-R and Mv-R-R with winter cover crops had more N_2_O emissions than Fa-R-R in both rice growing seasons (Fig. 3). N_2_O emission from paddy field is promoted with the amount of straw returning via increasing soil denitrification, which provides the soil microbial substrates and energy for soil nitrification and denitrification process [42]. Different ranking of treatments in N_2_O flux and total N_2_O emission might be related to the decomposition rates of winter crop species during the rice growing season. In the late rice growing season, the total N_2_O emissions of treatments Ry-R-R and Mv-R-R were significantly higher than Fa-R-R (*P<*0.05). This possibly results from that soil nitrification and denitrification process has been facilitated after the early rice straw returning through carbon and energy resource regulation (Table 1); a small amount of winter crop straw remains in the soil until the growing season of late rice; and tillage practice before late rice transplanting helps the incorporation of straws into soil, which may improve the soil nitrification and denitrification process.

### Global warming potentials of CH_4_ and N_2_O

Global warming potential can be used as an index to estimate the potential effects of different greenhouse gases on the global climate system. Bhatia et al. [5] estimated that GWPs of rice–wheat system increased by 28% on full substitution of organic N by chemical N. Zhu et al. [43] reported that the highest GWPs was found in Chinese milk vetch incorporation in double cropping rice system, which was 21–325% higher than the other three treatments. In this study, the GWPs of CH_4_, N_2_O or both had different orders. For a comprehensive consideration, GWPs of both CH_4_ and N_2_O is more important to assess the effect of a farming system on climate warming. Therefore, it is necessary to make a combined estimate of global warming effects of CH_4_ and N_2_O emitted from each treatment. Thus, we introduced the GWPs and per yield GWPs into this study for global warming calculations. Although the global warming effect of N_2_O is 12 times as large as that of CH_4_, CH_4_ emissions were nearly 370 times that of N_2_O, resulting in the majority of GWPs originating from CH_4_ (Table 3). Therefore, it is certain that the GWPs and per yield GWPs values for Ry-R-R and Mv-R-R were larger than Fa-R-R (*P<*0.05), due to their greater CH_4_ emissions. But the GWPs of CH_4_ and N_2_O and per yield GWPs of Mv-R-R was significantly lower than Ry-R-R (*P<*0.05). It should be mentioned that, the cultivation of ryegrass, Chinese milk vetch and its incorporation is a process involving C accumulation from the atmosphere to the soil, while the production of synthetic nitrogen fertilizer consumes fossil fuels that release C and contribute to greenhouse gas emissions. Therefore, we recommend Mv-R-R pattern in double cropping rice areas in the Middle and Lower reaches of Yangtze River in China, which correspond to Chinese milk vetch as winter cover crop + double rice.

## Conclusions

The emissions of CH_4_ and N_2_O from double cropping paddy fields were significantly enhanced by returning different winter cover crops. The effects on CH_4_ and N_2_O fluxes and emissions were different among treatments, and the emission characteristics varied greatly between early and late rice growing season. The orders of treatments were Ry-R-R>Mv-R-R>Fa-R-R for total emissions of CH_4_ and N_2_O during double rice seasons, and Ry-R-R>Mv-R-R>Fa-R-R for GWPs of total CH_4_ and N_2_O from double cropping paddy fields. Compared with Ry-R-R, Mv-R-R and Fa-R-R reduced CH_4_ emission during rice growing seasons. The GWPs (based on CH_4_ emission) under Mv-R-R and Fa-R-R was significantly (*P<*0.05) lower than Ry-R-R. Although the cumulative N_2_O emission under Ry-R-R and Mv-R-R were higher than that from Fa-R-R (*P<*0.05), GWPs of N_2_O was relatively low compared to that of CH_4_. The GWPs (based on CH_4_ and N_2_O) of Mv-R-R and Fa-R-R is lower than that of Ry-R-R (*P<*0.05). Meanwhile, the GWPs of CH_4_ and N_2_O and per yield GWPs of Mv-R-R was significantly lower than Ry-R-R (*P<*0.05). Thus, Mv-R-R is beneficial in GHG mitigation and it can be extended as an excellent cropping pattern in double rice cropped regions.
